# The socio-demographic patterning of sexual risk behaviour: a survey of young men in Finland and Estonia

**DOI:** 10.1186/1471-2458-9-256

**Published:** 2009-07-22

**Authors:** Minna Nikula, Mika Gissler, Vesa Jormanainen, Made Laanpere, Heikki Kunnas, Elina Haavio-Mannila, Elina Hemminki

**Affiliations:** 1 THL (National Institute for Health and Welfare), P.O. Box 30, 00271 Helsinki, Finland; 2 Finnish Defence Forces, P.O. Box 2, 15701 Lahti, Finland; 3 Department of Obstetrics and Gynaecology, University of Tartu, 50090 Tartu, Estonia; 4 Department of Mathematics and Statistics, University of Helsinki, P.O. Box 33, 00014 Helsinki, Finland; 5 Department of Sociology, University of Helsinki, P.O. Box 33, 00014 Helsinki, Finland

## Abstract

**Background:**

Sexually transmitted infections (STIs) among the youth are an increasing challenge for public health in Europe. This study provided estimates of men's (18–25 years) sexual risk behaviour and self-reported STIs and their socio-demographic patterning in Finland and Estonia; two countries that are geographically close, but have very different STI epidemics.

**Method:**

Nationally representative cross-sectional population surveys with comparable survey questions were used. Data from self-administered questionnaires for 1765 men aged 18–25 years in Finland (85% of the age cohort was included in the sampling frame, 95% of the sample responded) and 748 in Estonia, with a response rate of 43% respectively, were analysed. Socio-demographic patterning of multiple partners, condom use and self-reported STIs are presented was studied using multiple logistic regression analysis.

**Results:**

The main findings focus on associations found within each country. In Finland, higher age, low education and to a lesser extent relationship with a non-steady partner increased the likelihood of reporting multiple lifetime-partners, while in Estonia only higher age and low education revealed this effect. In relation to unprotected intercourse, in Finland, higher age, low education and relationship status with a steady partner increased the likelihood of reporting unprotected intercourse. In Estonia, the same was observed only for relationship status. In Finland the likelihood of self-reported STIs increased by older age and lower education and decreased by being with a non-steady partner, while in Estonia, a non-significant increase in self-reported STIs was observed only in the older age group.

**Conclusion:**

A clear socio-demographic patterning for sexual behaviour and self-reported STIs was revealed in Finland, but a less consistent trend was seen in Estonia. The findings of this study suggest that prevention strategies should focus in Finland on less educated singles and in Estonia on young men generally.

## Background

Sexually transmitted infections (STIs) among the youth are an increasing challenge for public health in Europe. Finland and Estonia, while geographically close, have very different STI epidemics [1,2]. After the restoration of independence in Estonia in 1991, the incidence of reported STIs began to increase [2-4]. Thereafter, many sexual health indicators, such as bacterial STIs, have improved, although HIV remains a serious health challenge in Estonia [2,5]. Finnish STI prevalence has traditionally been one of the lowest in Europe, but has gradually increased since the mid-1990s [1,6].

In 2006, the estimated rates of Syphilis and Gonorrhoea were over five times higher in Estonia than in Finland, whereas Chlamydia was 1.3 times higher in Finland than in Estonia [1,2,6]. The Estonian HIV prevalence is estimated to be 1.3%, which is over ten times the Finnish rate of 0.1% [2,6,7]. In Estonia HIV epidemic is been driven by injecting drug use (IDU), but is increasingly affecting drug user's partners via unprotected sex [2,6]. Whereas in Finland the outbreak among IDUs, although emerging around the same time, did not reach the extent of the Estonian epidemic, and sexual contact remains the most common way of transmission of HIV [2,6,7]. The youth aged under 25 have increasingly been affect by Chlamydia in Finland and by HIV in Estonia [1,2,6].

Estimating the impact of risk behaviour in public health draws on the current belief of a linkage between health and behaviour [8]. The collapse of the Soviet Union with an associated East-West health divide has encouraged comparative public health research to examine the socio-demographic patterning of risk behaviour in the two areas, which have very different public health platforms. Of particular interest in the case of Finland and Estonia is the potential dynamic link in the epidemiological development of STIs, particularly of HIV [9], given the geographical closeness and increasing cross-border traffic between the two countries.

Sexual risk behaviour studies among men became more common after the emergence of HIV epidemic in early 1990s [10]. Previously only one study has compared sexual behaviour among adult men in the 1990s in the Baltic region [11]. The present study focuses on young men's sexual risk behaviour and self-reported STIs as well as their association with socio-demographic characteristics in Finland and Estonia, including those of people of Russian ethnic origin living in Estonia.

## Methods

The data were derived from two nationally representative cross-sectional population surveys conducted in 2005 in Finland (n = 1765) and Estonia (n = 748). A more detailed description of the surveys has been presented previously [12,13].

The Finnish data were drawn from a health and lifestyle survey conducted in 2005 among men entering military service, which is mandatory for those aged 18–29 years. Some 85% of all men of this age group enter into military service, while less than 10% opt for non-military service, with the rest being unfit for service for health reasons (mainly due to physical conditions, followed by mental conditions). The majority of young men enter the service at the age of 18–19 years. Education is the most important determinant for the age of entrance to the service.

The Finnish defence forces is divided geographically into military units (brigades). A nationally representative number of brigades (regional location, large number of conscripts, no special or pre-selected troops) have administered a structured health and lifestyle survey annually since 1968. In each study brigade, every 4^th ^or 5^th ^person is selected to participate in the survey at the routine entrance medical examination procedure, which is carried out during the first two weeks of entry into the service. After the medical check-up, conscripts complete the questionnaires independently and anonymously, in a separate room under the guidance of the medical staff, and it is then returned by mail to the health division.

To derive comparable data for Estonia, a module of similar questions to those asked in Finland was added to a HIV KAP (Knowledge, Attitudes and Practice) -survey conducted in 2005 that included both men and women. A stratified (by three geographical regions and by two age groups in each region) random sample of people aged 19–29 years was drawn from the Estonian National Population Register. A self-administered anonymous questionnaire with one reminder was mailed.

In both surveys, similar questions on sexual behaviour and sexual health were combined with general health and lifestyle questions. The majority of the questions had multiple choice answers. All questions, answers and categorisations are shown in the Additional file 1.

Our analysis was restricted to men aged 18–25 in Finland and 19–25 in Estonia. The number of respondents in Finland was 1765, and the response rate among the selected conscripts was approximately 95%, considering that approximately 15% of the cohort does not enter the training and therefore were excluded from the sample frame. In Estonia the number of respondents was 748 with a response rate of 43% (after removing 10 questionnaires that were not suitable for analysis). The Estonian sample included 290 individuals of Russian ethnic origin. The data were analysed separately for each country. Only sexually active (ever engaged in sexual intercourse) respondents were included, except in the analysis of the proportions of those who were sexually active, those who had first sex intercourse under the age of 15 and those with STIs. Crude estimates were calculated for two age ranges for Finns, for those aged 18–25 and for those aged 19–25 (the latter to allow for a comparison with Estonia). Age adjustment was performed for the Estonian data using Finnish men as a reference population. Among Estonians, crude estimates were calculated separately for the two ethnic groups in Estonia, (those with Estonian and Russian nationality). Since the two ethnic groups had a similar age distribution, age-adjustment was not performed. A t-test for relative proportions was used to test for statistically significant differences between the two Estonian ethnic groups.

Logistic regression analysis was used to calculate odds ratios (OR) and 95% confidence intervals (CI) for associations of demographic factors (age, education and relationship status) with selected risk behaviour and self-reported STIs, separately for data from Finland and Estonia (Estonian and Russian ethnic groups living in Estonia together), and for the Russian ethnic group. Due to the small sample size of the Russian ethnic group, the regression analysis for self-reported STIs was conducted only with national samples.

## Results

The respondents in the Finnish sample were younger and more educated, and also included a higher proportion of individuals with a non-steady partner compared to respondents in Estonia (Additional file 2). The demographic composition of the samples of Estonians and those of Russian ethnic origin was similar.

The crude and age-adjusted prevalences of sexual behaviour are given in Table 1. One third of Finns and slightly less than a quarter of Estonians reported having had six or more lifetime sex partners (multiple lifetime-partners) in both countries and about one fifth reported having had three or more sex partners in the past year. In relation to condom use, approximately half of men reported using condoms during their last sexual intercourse.

**Table 1:** Crude and age-adjusted percentage of selected behaviour and self-reported STIs, men 18–25, Finland and Estonia, 2005

	Finland		Estonia^a^	Estonia E^a^	Estonia R^a^
	%		Adjusted %^b^	Crude %^c^	

Age	18–25	19–25	19–25	19–25	19–25

n	1765	1464	748	458	290

Sexually active ^d^	75	77	84	89	88

First sex < 15 years^d^	13	14	10	10	13

3+ sex partners, past year^e^	20	20	17	17	20

6+ sex partners, lifetime^e^	31	33	22	31	34

Condom use, last intercourse^e^	51	49	52	41	42

Reason for using condom ^e, f^					

STI protection	17	18	13	11	11

Avoiding pregnancy	25	24	58	59	57

Both	47	47	28	29	31

Other reasons	11	11	1	1	1

Self-reported STI, ever ^d, g^	3	3	5	8	7

^a^Estonia = All Estonians including those with Russian ethnic origin, Estonia E = Only those with Estonian ethnic origin, Estonia R = Only those with Russian ethnic origin

^b ^Age-adjusted by using Finnish men as reference population

^c ^Differences between Estonia E and Estonia R were statistically non-significant

^d ^Numerator includes all respondents

^e ^Numerator includes all sexually active respondents

^f ^Question formulation differed in Finland and Estonia, see Additional file 1.

^g ^STI = Sexually Transmitted Infection = Chlamydia, Gonorrhoea or Syphilis

The reasons for using a condom differed. Estonians used a condom primarily to avoid an unwanted pregnancy, whereas most Finns reported using a condom for two reasons, preventing STIs and unwanted pregnancy. A separate cross-tabulation (not shown) aligning partner type with condom use during last intercourse revealed that some 30% of Finns and Estonians did not use a condom even if the partner was a previously unknown person.

All differences in sexual behaviour as well as self-reported STIs between Russian and Estonian ethnic groups were minor and statistically non-significant (Table 1).

In relation to reporting multiple lifetime-partners, when differences were observed they were of a similar direction in Finland and Estonia; higher age, low education and relationship with a non-steady partner increased the likelihood of engagement with multiple lifetime-partners (Table 2). In Finland age, education and to a lesser extend relationship with a non-steady partner had a statistically significant association with reporting multiple lifetime-partners, while in Estonia only age and education revealed this effect. The predictive values of age and education for multiple partners were almost equally strong in Finland and Estonia. However, a relationship with a non-steady partner increased statistically significantly the likelihood of engagement with multiple lifetime-partners, a finding observed in Finland but not among Estonians. In respect to the Russian ethnic group, only lower education was statistically significantly associated with multiple partners.

**Table 2:** Multiple lifetime-partners (6+) by selected socio-demographic and sexual behaviour variables, adjusted odds ratios and their 95% confidence intervals (CI), sexually active men in Finland (18–25 years) and Estonia (19–25 years), 2005^a^

		Finland			Estonia^b^			Estonia R^b^	
		n = 1313			n = 637			n = 239	

	%	OR	95% CI	%	OR	95% CI	%	OR	95% CI

*Age*^*c*^									

18	20	1.00							

19	24	1.25	(0.82 1.91)	19	1.00		29	1.00	

20–25	41	2.53	(1.68 3.83)	33	2.57	(1.41 4.69)	35	1.77	(0.71 4.39)

*Education*^*c*^									

University & High school	23	1.00		27	1.00		29	1.00	

Vocational	34	1.63	(1.23 2.14)	27	1.08	(0.70 1.66)	28	1.18	(0.56 2.50)

Comprehensive	43	2.16	(1.54 3.03)	44	2.66	(1.62 4.37)	51	3.33	(1.40 7.92)

*Relationship status*^*c, d*^									

Steady	27	1.00		31	1.00		30	1.00	

Non-steady	36	1.49	(1.16 1.91)	31	1.05	(0.69 1.59)	41	1.71	(0.88 3.32)

*First sex < 15 years*^*e*^									

No	22	1.00		27	1.00		28	1.00	

Yes	71	9.56	(6.73 13.59)	69	5.59	(2.89 10.81)	76	7.44	(2.70 20.49)

^a ^All sexually active respondents

^b ^Estonia = All Estonians including those with Russian ethnic origin, Estonia R = Only those with Russian ethnic origin

^c ^Adjusted for all variables except for first sex < 15 years

^d ^Relationship status = current relationship status

^e ^Adjusted for all variables

When adjusted for all socio-demographic factors, intercourse before the age 15 years increased the likelihood of engagement with multiple lifetime-partners and revealed clearly marked differences in reporting multiple lifetime-partners in each of the three groups (Table 2).

In relation to unprotected intercourse (non-use of condom), when differences were observed they were of a similar direction in both study countries; higher age, lower education and being in a steady relationship increased the likelihood of reporting unprotected intercourse (Table 3). In Finland, those reporting unprotected intercourse showed statistically significant differences for age, education and relationship status. In Estonia, the same was observed only for relationship status, while statistically non-significant differences were found by age, though educational differences were absent. Relationship status (being in a steady relationship) was the strongest predictor for unprotected intercourse, being almost equally strong in Finland than in Estonia as well as among the Russian ethnic group. A clear difference between the two Estonian samples was observed, with age being a stronger correlate for reporting unprotected intercourse among men of Russian ethnic origin. Early age at first sexual intercourse revealed statistically significant differences among those reporting unprotected intercourse only in Finland. However, differences of a similar direction but statistically non-significant were also observed in Estonia and among the Russian ethnic group.

**Table 3:** Unprotected last intercourse^a ^by selected socio-demographic and sexual behaviour variables, adjusted odds ratios and their 95% confidence intervals (CI), sexually active men in Finland (18–25 years) and Estonia (19–25 years), 2005^b^

		Finland			Estonia^b^			Estonia R^b^	
		n = 1313			n = 637			n = 239	

	%	OR	95% CI	%	OR	95% CI	%	OR	95% CI

*Age*^*d*^									

18	37	1.00							

19	50	1.81	(1.24 2.65)	45	1.00		32	1.00	

20–25	52	1.92	(1.31 2.81)	61	1.59	(0.90 2.83)	62	2.72	(1.02 7.25)

*Education*^*d*^									

University & High school	44	1.00		60	1.00		56	1.00	

Vocational	53	1.50	(1.15 1.97)	57	0.90	(0.58 1.39)	55	1.04	(0.49 2.21)

Comprehensive	50	1.35	(0.95 1.91)	62	1.14	(0.67 1.92)	65	1.81	(0.72 4.57)

*Relationship status*^*d, e*^									

Steady	62	1.00		67	1.00		68	1.00	

Non-steady	30	0.25	(0.19 0.32)	36	0.29	(0.19 0.45)	31	0.24	(0.12 0.50)

*First sex < 15 years*^*f*^									

No	44	1.00		58	1.00		56	1.00	

Yes	72	3.26	(2.35 4.52)	68	1.54	(0.82 2.89)	66	1.78	(0.72 4.42)

^a ^Intercourse without condom

^b ^All sexually active respondents

^c ^Estonia = All Estonians including those with Russian ethnic origin, Estonia R = Only those with Russian ethnic origin

^d ^Adjusted for all variables except for first sex < 15 years

^e ^Relationship status = current relationship status

^f ^Adjusted for all variables

In Finland the likelihood of self-reported STIs increased statistically significantly in the older age group and decreased by being with a non-steady partner (Table 4). Low education also increased the likelihood of self-reported STIs in Finland, but its effect was statistically non-significant. In Estonia, a non-significant increase in self-reported STIs was observed in the older age group, whereas differences by education or relationship status were not found. In both countries STIs also statistically significantly increased to a similar extent among those who reported unprotected intercourse and multiple lifetime-partners.

**Table 4:** Self-reported STIs by selected socio-demographic and sexual behaviour variables, adjusted odds ratios and their 95% confidence intervals (CI), men in Finland (18–25 years) and Estonia (19–25 years), 2005^a^

		Finland			Estonia^b^	
		n = 1765			n = 748	

	%	OR	95% CI	%	OR	95% CI

*Age*^*c*^						

18	1	1.00				

19	2	1.65	(0.47 5.81)	5	1.00	

20–25	5	4.07	(1.21 13.62)	9	1.88	(0.72 4.92)

*Education*^*c*^						

University & High school	2	1.00		*9*	1.00	

Vocational	3	1.34	(0.69 2.61)	7	0.85	(0.44 1.65)

Comprehensive	5	1.68	(0.77 3.66)	8	1.05	(0.48 2.28)

*Relationship status*^*c, d*^						

Steady	4	1.00		*8*	1.00	

Non-steady	2	0.53	(0.29 0.97)	8	0.96	(0.52 1.77)

*Multiple lifetime-partners (6+)*^*e*^						

No	2	1.00		6	1.00	

Yes	9	4.77	(2.45 9.28)	15	3.11	(1.61 6.02)

*Unprotected last intercourse*^*e*^						

No	2	1.00		7	1.00	

Yes	5	2.21	(1.09 4.51)	12	2.17	(1.05 4.49)

^a ^All respondents

^b ^Estonia = All Estonians including those with Russian ethnic origin

^c ^Adjusted for all variables except for multiple lifetime partners and unprotected intercourse

^d ^Relationship status = current relationship status

^e ^Adjusted for age, education and relationship status

The odds ratios presented in Tables 2, 3 and 4 for Finland were derived using '18 years of age' as a reference category; however, setting an age of 19 years as a reference group (as in Estonia) did not change the level of significance in the association of age with the studied indicators.

## Discussion

This study provided estimates of men's sexual risk behaviour and self-reported STIs and their socio-demographic patterning in two countries with different STI epidemics. The most important finding of our study revealed a clear socio-demographic patterning for sexual risk behaviour and self-reported STIs in Finland, but a less consistent trend in Estonia. In both countries the likelihood of reporting multiple lifetime-partners increased statistically significant by higher age (20–25 years), lower education (comprehensive) and in Finland additionally by a non-steady relationship status. The likelihood of unprotected intercourse was statistically significantly higher in a non-steady relationship in both countries, while additionally among those with higher age and lower education (vocational) in Finland. The association of self-reported STIs was statistically significantly more common in the oldest age group (20–25 years) and in a steady relationship in Finland, while in Estonia these effects were absent.

There are a few methodological issues worth noting. The strength of our study is a high response rate in Finland, enabled by the mandatory military service that provides for a unique opportunity to conduct surveys on young men who, compared to women, are hard to reach and poorly motivated to provide reliable survey information [14,15]. A major limitation of the study is the difference in response rates in the two countries. Although the response rate was high in Finland, 15% of the age cohort is exempted from military training [16]. We know that non-respondents of the military or civil service are less educated and more likely to smoke, use alcohol or have ever used of drugs, but we do not know how the sexual behaviour of those exempted differs from the rest who enter the service [16]. Sexual orientation was not asked in the military context or in the Estonian survey. In Finland a majority of men who have sex with men opt for military training (70%) [17]. The characteristics of the high number of non-respondents in Estonia are unknown; however, based on documentation from a variety of sexual behaviour studies, those with conservative or normative lifestyles are less likely to participate in self-reported sexual surveys [18-20]. On the other hand, it is likely that non-respondents also included people who did not participate for social reasons, such as addiction to alcohol or drugs, which are known to correlate with sexual risk behaviour [12].

Other limitations for comparability are the differences in the setting (military and household) and purpose (general health survey and HIV KAP-survey) of the two surveys, which may have effected selection and reporting biases in any direction. Furthermore the small number of comparable indicators used as well as the difference in the question design could have further jeopardised the comparability and also the validity of the findings. Recall and reporting biases on retrospective or sensitive data as well as adverse experiences may have led to under- or overestimates [15]. In a survey among men, over-reporting is likely to have occurred for the number and type of sexual partners and for condom use [15]. Also non-awareness of STI diagnosis could have biased self-reporting of STIs. However, a European cross-national survey which converged the sex ratio and distribution of the four major STIs with epidemiological surveillance concluded that errors did not contribute a major bias in STI reporting [10].

It is likely, however, that reporting biases have shaped the two national surveys alike and thus does not interfere with comparability. Finally, the small sample size of those of Russian ethnic origin did not allow all analyses to be made by ethnic group in Estonia. The net effect of these limitations has the potential to lead either to an under- or overestimate in prevalence levels of behaviour [15]. Therefore we focus mainly on associations found within each country.

Studies of men's sexual behaviour in Finland as well as in the Baltic region are scarce and they bear similar limitations to our study [11]. However our results corroborate earlier data derived from an Estonian population-based HIV survey (aged 19–25, response rate 41%) [21], and Finnish national school health surveys (men aged 17–18) [22], military health surveys (men aged 18–25, response rate 95%) [12] and national health surveys (men aged 18–24, response rate 61%) [23]. Similar prevalences were found in the earlier research for early age at first sexual intercourse, partnering patterns and condom use among young men, One previous comparable population-based survey on men's sexual behaviour in the Baltic sea area [11] conducted in 1999–2000 (response rates Finland 46%, Estonia 41%) found that attitudes towards sexual relationship outside of steady dating were more positive among Estonians, that their average age of sexual debut was 1–1.5 years older than in Finland, that the average number of lifetime partners was lower, and that condom use at last intercourse was also lower (data of a comparative age group to our study was provided by Elina Haavio-Mannila, 2008) [11]. Haavio-Mannila et al. associated this with the delayed western influence of sexual liberation in Estonia [11].

The reasons that the men surveyed reported for using a condom were different in Finland compared to Estonia. This suggests that young men in Estonia perceive condoms as a contraceptive method rather than a means to prevent STIs. Since the vast majority of young adults use either condoms or birth control pills to prevent unwanted pregnancies [11,22,24], our findings are likely to reflect the fact that condoms are more commonly used for contraception in Estonia than in Finland. Although we can not know the effect of a slight difference in question design, it is not likely that it would fully explain this finding.

We found that the socio-demographic patterning of multiple lifetime-partners was most consistent of the measured indicators between Finland and Estonia. In Finland, the relationship with a non-steady partner predicted engagement in multiple lifetime-partners. A similar effect of relationship status or marital status on partnering patterns has been reported widely in the EU region, and therefore one might have expected to find this correlation across the study countries [25-27]. However, surprisingly, in Estonia, relationship status did not correlate with the reporting of multiple lifetime-partners. One explanation for our finding could be the higher tolerance for casual sexual contacts outside a long-term partnership, which has also been previously observed in Estonia [11].

In contrast to the inconsistent association of relationship status with multiple lifetime-partners found in Finland and Estonia, the association of relationship status with those reporting unprotected intercourse was nevertheless similarly strong in both countries, as well as among the Russian ethnic group. This is a common finding and relates to the fact that, in general, a condom is the first and most common method for contraception among young people, who constitute a major part of those in a non-steady relationship (even when results are age adjusted) [25-27].

Furthermore, while relationship-type and age-related differences in unprotected intercourse were in a similar direction, educational differences in unprotected intercourse were observed only in Finland. Numerous studies on the socio-demographic patterning of men's sexual risk behaviour, mortality and morbidity point to an accumulation of health problems for men with a low educational level in post-Soviet countries [9,28]. On the other hand, some findings suggest that a high level of education might be a stronger protective factor for health in a stable market economy than in a post-Soviet society [29,30]. It remains to be seen whether the rapid liberalization of the Estonian society is actually flattening out the educational differences in sexual risk behaviour.

Although the differences in prevalence for self-reported STIs between Finland and Estonia were minor and susceptible to survey limitations, they do echo similar differences found in STI epidemics through case surveillance systems [1,2]. Our results on the association of other well-known risk factors with self-reported STIs support what has been widely established i.e. the fact that a high number of sexual partners and unprotected intercourse are key determinants for STIs [15,31]. The association of multiple partners and unprotected intercourse with self-reported STIs was clear and similar in Finland as well as in Estonia.

## Conclusion

While there are similarities between Estonia and Finland, there are also significant differences. In Estonia, HIV infection is been driven by injecting drug use (IDU) [2], and in Finland via sexual contacts. However, in Estonia HIV is increasingly being transmitted from IDUs to their sexual partners via unprotected sex [2], and the crucial question is to what extent this is going to affect the general population. In Finland currently over one third of Finns obtain HIV from unprotected sex while travelling [1,6]. Besides the different driving factors of the HIV epidemic, the important question is whether the Estonian epidemic is going to affect the Finnish HIV incidence rate, since Estonia is the second most popular target country for Finnish travellers, and Finland is the most preferred destination for Estonian labor migrants due to the relatively low language barrier [32]. While the current dynamic of enhanced economic integration, increased migration, exchanges of labour and ease of travel between Finland and Estonia is conducive to impacting the STI epidemic, it is also an opportunity for strengthening cross-border co-ordination in STI prevention.

Cross-national behavioural surveillance can facilitate understanding the factors behind STI epidemics. The findings of this study suggest that prevention strategies should focus in Finland on less educated singles and in Estonia on young men generally. Moreover, there is clear benefit in further collaboration in comparative behavioural research and surveillance systems between the two countries, using innovative methods to increase response rates among young men, such as internet-assisted administration methods. Other more cost effective ways such as including a module of standardised questions on sexual health in a general health survey should be explored, as well as conducting surveys among cross-travellers and high risk groups, such as those who engage in commercial sex. To gain further understanding of the validity of self-reports in such surveys, a smaller scale STI clinic based surveys with non-invasive STI test could be useful.

## Competing interests

The authors declare that they have no competing interests.

## Authors' contributions

MN participated in the institutional coordination, survey question formulation, design of the analytical approach and manuscript drafting. MG and ML participated in the manuscript revision and supervision. VJ participated in the institutional coordination and manuscript revision. E H-M participated in the supervision and manuscript revision. EH participated in the design of the analytical approach, supervision and manuscript revision. HK performed the statistical analysis. All authors read and approved the final manuscript.

## Pre-publication history

The pre-publication history for this paper can be accessed here:



## Supplementary Material

Additional file 1 **Questions, answers and categorisations used in the study**. Age, education and relationship status were used to characterize the population groups. The categorization is shown in Table 1 Click here for file

Additional file 2 **Background factors, men in Finland and Estonia, 2005**. Age, Education and relationship statuses of men in Finland and Estonia, 2005 Click here for file
