# Supplementary material for: The socio-demographic patterning of sexual risk behaviour: a survey of young men in Finland and Estonia
Source: BMC Public Health. 2009 Jul 22;9:256. doi: 10.1186/1471-2458-9-256 (PMC2718888; doi:10.1186/1471-2458-9-256)
Supplement: Additional file 1 — Questions, answers and categorisations used in the study. Age, education and relationship status were used to characterize the population groups. The categorization is shown in Table 1 [file 1471-2458-9-256-S1.doc]

**Additional file 1.** Questions, answers and categorisations used in the study

Age, education and relationship status were used to characterize the population groups. The categorization is shown in Table 1.

**Age**

"How old are you?" Open-ended question.

Categorization is shown in Table 1

**Education**

In Finland education was assessed through a question with the following 6 answer alternatives;

a) only comprehensive up to 9 years,

b) high school with 12 years completed,

c) vocational with 12 years completed, d) technical from 12 upwards completed,

e) university from 12 years upwards completed,

f) other,

**Education**

In Estonia education was assessed through a question with the following 7 answer alternatives;

a) only comprehensive up to 9 years,

b) high school ongoing,

c) high school with 12 years completed,

d) vocational ongoing,

e) vocational up to12 years completed,

f) higher technical or university ongoing,

g) higher technical or university from 12 years upwards completed.

Categorized by completed years of education into the following three groups;

i) comprehensive, at least 9 years completed,

ii) vocational, at least 12 years or vocational education completed,

iii) university and high school = at least 12 years of high school completed or, university or higher technical ongoing or completed.

**Ethnicity**

The ethnicity was identified by asking: "What is your nationality?"

a) Estonian,

b) Russian

c) other

**Partnership status**

Current partnership status was assessed in Finland by the following two questions:

"What is your current marital status?"

a) unmarried,

b) engaged or cohabiting,

c) married

d) divorced or widowed

"Was your partner with whom you had your last sexual intercourse?"

a) your spouse or steady partner,

b) some else's spouse or partner,

c) single person who you knew,

d) single person who you did not know,

e) prostitute, and

f) I don't know.

Categorized;

i) steady partner = engaged, cohabiting, married, spouse or steady partner and

ii) non-steady = others.

In Estonia the question was:

"What best describes your partnership status during the past 4 weeks?"

a) I live with my steady partner,

b) I have a steady partner who does not live with me,

c) I have a non-steady partner/s,

d) I do not have a steady, or a non-steady partner and

e) I have not started sexual life yet.

Categorized;

i) steady partner = lives or does not live with a steady partner and

ii) non-steady partner = others.

**Questions on sexual behaviour had the following wording and categorization:**

Have you ever engaged in sexual intercourse?

a) yes

b) no

c) I do not want to answer (Estonia only)

Categorized;

0 = no

1 = yes

"At what age did you have your first sexual intercourse?" Open-ended question.

Categorised;

i) < 15 years,

ii) ≥ 15 years.

"With how many different persons altogether you have had sexual intercourse during your lifetime / during the past 12 months?" Open ended question.

Categorised;

i) less than 6,

ii) 6 or more.

"Did you use a condom during your last sexual intercourse?"

a) yes,

b) no and

c) don't know.

Categorized;

i) yes,

ii) no.

Has your physician ever diagnosed any of the following diseases for you?

13 alternatives for different STIs, each alternative answered with either

a) yes,

b) no or

c) don't know.

Categorised;

i) Chlamydia, Syphilis or Gonorrhoea,

ii) no Chlamydia, Syphilis or Gonorrhoea

Reasons for using a condom were assessed by the following question in Finland:

"If you used a condom in your last intercourse, why did you use it (you can choose several alternatives)?"

a) my partner wanted it,

b) for curiosity,

c) because my friends use one,

d) I heard that condoms protects against STIs,

e) to prevent unwanted pregnancy,

f) I wanted to protect myself because I did not know my partner.

Categorized;

i) prevent STIs = alternative d but not e,

ii) prevent unwanted pregnancy = alternative e but not d,

iii) prevent both STIs and unwanted pregnancy = both alternatives d and e.

iv) other reasons = a or b, or c, or f but not d or e

In Estonia two questions were used:

"If you have used a condom currently or during the last 12 months why have you used it (you can choose only one alternative)?"

a) I have not had sexual intercourse,

b) mainly to prevent unwanted pregnancy,

c) mainly to prevent STIs,

d) equally for both of the last two reasons,

e) other and

f) I have not used a condom.

"Did you use a condom during your last sexual intercourse?"

a) yes,

b) no and

c) don't know.

Categorized;

i) to prevent STIs = alternative c and a1

ii) prevent unwanted pregnancy = alternative b and a1

iii) both = d and a1

iv) other = e and a1
