# Supplementary material for: The socio-demographic patterning of sexual risk behaviour: a survey of young men in Finland and Estonia
Source: BMC Public Health. 2009 Jul 22;9:256. doi: 10.1186/1471-2458-9-256 (PMC2718888; doi:10.1186/1471-2458-9-256)
Supplement: Additional file 2 — Background factors, men in Finland and Estonia, 2005. Age, Education and relationship statuses of men in Finland and Estonia, 2005 [file 1471-2458-9-256-S2.doc]

**Additional file 2.** Background factors, men in Finland and Estonia, 2005

|  |  |  | Finland |  | Estonia E^a^ |  | Estonia R^a^ |
| --- | --- | --- | --- | --- | --- | --- | --- |
|  |  |  | n= 1765 |  | n= 458 |  | n= 290 |
|  |  |  | % |  | % |  | % |
| **Age** |  |  |  |  |  |  |  |
| 18 |  |  | 16 |  |  |  |  |
| 19 |  |  | 44 |  | 16 |  | 16 |
| 20 |  |  | 30 |  | 20 |  | 18 |
| 21 |  |  | 5 |  | 15 |  | 19 |
| 22 |  |  | 2 |  | 16 |  | 17 |
| 23-25 |  |  | 3 |  | 33 |  | 30 |
| **Education** | |  |  |  |  |  |  |
| Comprehensive | |  | 14 |  | 23 |  | 21 |
| Vocational | |  | 36 |  | 46 |  | 50 |
| University & High school | | | 50 |  | 31 |  | 29 |
| **Stable relationship** | |  |  |  |  |  |  |
| No |  |  | 57 |  | 36 |  | 38 |
| Yes |  |  | 43 |  | 64 |  | 62 |
| ^a^ Estonia E = Only those with Estonian ethnic origin, Estonia R = Only those with Russian ethnic origin | | | | | | | |
|
